# A neuropathological cell model derived from Niemann−Pick disease type C patient-specific iPSCs shows disruption of the p62/SQSTM1−KEAP1−NRF2 Axis and impaired formation of neuronal networks

**DOI:** 10.1016/j.ymgmr.2021.100784

**Published:** 2021-07-24

**Authors:** Ryo Saito, Takashi Miyajima, Takeo Iwamoto, Chen Wu, Ken Suzuki, Mohammad Arif Hossain, Miyo Munakata, Takumi Era, Yoshikatsu Eto

**Affiliations:** a Advanced Clinical Research Center, Southern Tohoku Research Institute for Neuroscience, 255 Furusawa, Asao-ku, Kawasaki 215-0026, Japan; b Core Research Facilities for Basic Science, The Jikei University School of Medicine, 3-25-8 Nishishinbashi, Minato-ku, Tokyo 105-8461, Japan; c Rare disease research center, AnGes Inc., 127 Furusawa, Asao-ku, Kawasaki 215-0026, Japan; d Advanced Clinical Research Center, Southern Tohoku Research Institute for Neuroscience, 7-115 Yatsuyamada, Koriyama, 963-8563, Japan; e Department of Cell Modulation, Institute of Molecular Embryology and Genetics, Kumamoto University, 2-2-1 Honjo, Chuo-ku, Kumamoto 860-0811, Japan; f The Jikei University School of Medicine, 3-25-8 Nishishinbashi, Minato-ku, Tokyo 105-8461, Japan.

**Keywords:** Niemann−Pick disease type C, Lysosomal storage disorder, Induced pluripotent stem cells, Cell-based neuropathological model, Neuronal network density, p62/SQSTM1−KEAP1−NRF2 axis

## Abstract

Niemann−Pick disease type C (NPC) is a rare neurodegenerative disorder caused by a recessive mutation in the *NPC1* or *NPC2* gene, in which patients exhibit lysosomal accumulation of unesterified cholesterol and glycolipids. Most of the research on NPC has been done in patient-derived skin fibroblasts or mouse models. Therefore, we developed NPC patient neurons derived from induced pluripotent stem cells (iPSCs) to investigate the neuropathological cause of the disease. Although an accumulation of cholesterol and glycolipids, which is characteristic of NPC, was observed in both undifferentiated iPSCs and derived neural stem cells (NSCs), we could not observed the abnormalities in differentiation potential and autophagic activity in such immature cells. However, definite neuropathological features were detected in mature neuronal cells generated from NPC patient-derived iPSCs. Abnormal accumulation of cholesterol and other lipids identified by lipid droplets and number of enlarged lysosomes was more prominent in mature neuronal cells rather than in iPSCs and/or NSCs. Thin-sectioning electron microscopic analysis also demonstrated numerous typical membranous cytoplasmic bodies in mature neuronal cells. Furthermore, TUJ1-positive neurite density was significantly reduced in NPC patient-derived neuronal cells. In addition, disruption of the p62/SQSTM1−KEAP1−NRF2 axis occurred in neurons differentiated from NPC patient-derived iPSCs. These data indicate the impairment of neuronal network formation associated with neurodegeneration in mature neuronal cells derived from patients with NPC.

## Highlights

• Niemann−Pick disease type C patient-derived neurons showed pathological features
• Lipid droplets and lysosomes accumulated at high levels in patient's cells
• Patient-derived neurons showed defective neuronal network formation
• Disruption of the p62/SQSTM1−KEAP1−NRF2 axis occurred in patient-derived neurons

## 1 Introduction

Human induced pluripotent stem cells (iPSCs) are now becoming an effective platform for regenerative therapy, disease modeling, and drug discovery. With the development of iPSC technology, disease-specific iPSCs have become accessible as they are easily generated from patients' somatic cells including their fibroblasts and peripheral blood mononuclear cells [[1], [2], [3], [4]]. By providing appropriate developmental stimuli, these cells can be differentiated into a variety of other cells. It has recently been demonstrated that the phenotype of a disease can be faithfully reproduced by differentiating disease-specific iPSCs into target cells and/or tissues [[5], [6], [7], [8]]. Given that the biological samples obtained from patients suffering from neurological diseases, e.g., neurodegenerative disorders and psychiatric disorders, have typically been limited to serum, plasma, and frozen/formalin-fixed paraffin embedded-postmortem brain samples, the neurons differentiated from disease-specific iPSCs are considered to be important living samples for pathological analysis and development of new therapies [[9], [10], [11], [12]].

Niemann−Pick disease type C (NPC) is a rare neurodegenerative disorder caused by autosomal recessive loss-of-function in either the *NPC1* (95% of cases) or *NPC2* (5% of cases) genes [13,14]. The clinical hallmarks of NPC include a combination of dysphagia, cerebellar ataxia, dystonia, and vertical supranuclear gaze palsy. Patients with NPC may also present with a range of psychiatric symptoms including cognitive decline, schizophrenia-like psychosis, and mood disorders. NPC is genetically heterogenous with approximately 400 and 23 pathogenic mutations in *NPC1* and *NPC2*, respectively. These pathogenic mutations lead to lysosomal accumulation of unesterified cholesterol, sphingolipids, and glycolipids in the cells of patients [[15], [16], [17]]. Although the function of NPC1 has yet to be fully revealed, both NPC1 and NPC2 are believed to act in the transport of various lipid species from late endosomes and lysosomes. Mitochondrial cholesterol accumulation in NPC1-deficient cells has also been reported, suggesting that a relationship exists between mitochondrial dysfunction and the pathogenesis of NPC [18]. In support of this hypothesis, mitochondrial abnormalities and related brain dysfunction have been observed in NPC1-deficient mice [19]. Although the involvement of altered intracellular cholesterol homeostasis and mitochondrial dysfunction in the neuropathology of NPC has been documented, the molecular mechanisms underlying neurodegeneration remain unclear.

Autophagy is an evolutionarily conserved process in which damaged organelles and unwanted/mutated proteins, encapsulated in autophagosomes, are degraded by fusion with lysosomes to maintain intracellular homeostasis. p62/SQSTM1 plays a central role in selective autophagy involving the clearance of ubiquitinated substrates and dysfunctional mitochondria [20,21]. Several studies have shown that endosomal and autophagic pathway dysfunction is strongly associated with the progression of neurodegenerative disorders, such as Alzheimer's disease, Parkinson's disease, and amyotrophic lateral sclerosis, as well as inherited lysosomal storage disorders characterized by the intralysosomal buildup of partially degraded metabolites [[22], [23], [24]]. Importantly, recent studies involving NPC patient-derived fibroblasts, human embryonic stem cell (hESC)-derived neurons with NPC1 knockdown, and *Npc1*^−/−^ mouse models have revealed that autophagic dysfunction is part of NPC pathogenesis, i.e., accumulation of autophagosomes, characteristic autophagic multivesicular structures, and lysosomes has been observed in the brain of *Npc1*^−/−^ mice, neural stem/progenitor cells differentiated from NPC patient-derived iPSCs, and NPC patient-derived fibroblasts [[25], [26], [27], [28]]. Although NPC1-deficient cells and *Npc1*^−/−^ mice have been widely used in biological assessments, examples of pathological analysis of cells and/or mice with heterogeneous *NPC1* mutations, such as those in actual patients, are currently lacking [29]. Furthermore, in recent studies using iPSCs derived from patients with NPC, analysis has been limited to differentiation into hepatocytes and neural stem/progenitor cells [28,[30], [31], [32]]; thus, the pathological mechanisms in mature neurons have not yet been clarified. In the present study, we comparatively analyze pathological and molecular changes in various differentiation states [i.e., undifferentiated iPSCs, neural stem cells (NSCs), and neurons] using iPSCs derived from patients with NPC and healthy controls.

## 2 Materials and methods

### 2.1 Undifferentiated human iPSCs culture

The human iPSC lines 201B7 and Nips-B2 were used as healthy control iPSC lines. The NPC patient-derived human iPSC lines NPC5–1 and NPC6–1 were used as the NPC iPSC lines; NPC5–1 and NPC6–1 carry heterozygous mutations p.S667L/p.C1161Y and p.F194*/p.Y1088C at the NPC1 protein, respectively. The basic information on the origin of all human iPSCs (i.e., medical history, gender, age, tissue, reprogramming factors, and vector type) is summarized in Table 1. All human iPSCs were maintained in StemFit AK02N medium (Ajinomoto, Tokyo, Japan) with penicillin−streptomycin solution (1×; FUJIFILM Wako Pure Chemical Corporation, Osaka, Japan). When the culture reached approximately 80% confluency, cells were dissociated using TrypLE Select Enzyme (0.5×; Thermo Fisher Scientific, MA, USA) in Dulbecco's phosphate-buffered saline (DPBS) (−) and seeded at 1.0 × 10^4^ cells/well on iMatrix-511 silk (0.375 μg/cm^2^; Nippi, Tokyo, Japan)-coated six-well plates in the presence of 10-μM Y-27632 (FUJIFILM Wako Pure Chemical Corporation). For routine maintenance, fresh medium without Y-27632 was changed on days 1, 3, 5, 6, and 7, and cells were passaged on day 8.

**Table 1:** Basic information on human induced pluripotent stem cells.

Cell Name	Medical history	Gender	Age	Tissue	Reprogramming factors	Vector type
201B7	No information	Female	36 years	Skin fibroblast	Oct3/4, Sox2, Klf4, c-Myc	Retrovirus
Nips-B2	Atopic asthma	Female	43 years	Nasal epithelial cells	Oct3/4, Sox2, Klf4, c-Myc	Sendai virus
NPC5–1	Niemann-Pick disease type C	Male	16 years	Skin fibroblast	Oct3/4, Sox2, Klf4, c-Myc	Sendai virus
NPC6–1	Niemann-Pick disease type C	Female	6 years	Skin fibroblast	Oct3/4, Sox2, Klf4, c-Myc	Sendai virus

### 2.2 Neural induction of iPSCs and NSC expansion

Human iPSCs were seeded at 2.0 × 10^5^ cells/well on Geltrex LDEV-Free hESC-qualified Reduced Growth Factor Basement Membrane Matrix (Thermo Fisher Scientific)-coated six-well plates in the presence of 10-μM Y-27632. Approximately 24 h after seeding, culture medium was switched to PSC Neural Induction Medium (Thermo Fisher Scientific) containing Neurobasal Medium and Neural Induction Supplement. Neural Induction Medium was changed every other day from day 0 to day 6 of neural induction. From day 4 of neural induction, media volume was increased 2-fold. At day 7 of neural induction, primitive NSCs were dissociated using StemPro Accutase Cell Dissociation Reagent (Thermo Fisher Scientific) and then seeded at 1.0 × 10^6^ cells/well on Geltrex-coated six-well plates in Neural Expansion Medium containing 50% Neurobasal Medium, 50% Advanced DMEM/F12, Neural Induction Supplement, 0.25% AlbuMAX I Lipid-Rich bovine serum albumin (BSA), 1× penicillin−streptomycin solution, and 10-μM Y-27632. Culture medium without Y-27632 was changed every other day until NSCs reached confluence.

### 2.3 Differentiation of expanded NSCs to neurons

Expanded NSCs were maintained in Neural Expansion Medium and treated with 20-μM DAPT (a γ-secretase inhibitor; Merck Millipore, MA, USA) for 2 days prior to the start of neuronal differentiation. The DAPT-treated NSCs were dissociated using StemPro Accutase Cell Dissociation Reagent and seeded at 5.0 × 10^5^ cells/well on 0.05% polyethylenimine (Merck Millipore) and 0.5-μg/cm^2^ iMatrix-511 silk-coated six-well plates. These NSCs were differentiated in Neuronal Differentiation Medium containing Neurobasal Medium, 1.0× B-27 Supplement Minus Vitamin A (Thermo Fisher Scientific), 1.0× GlutaMAX-I (Thermo Fisher Scientific), 200-μM L-ascorbic acid (Merck Millipore), 0.25% AlbuMAX I Lipid-Rich BSA, and 1× penicillin−streptomycin solution in addition to 20-μM DAPT, 2.0-μM PD0332991 (a CDK4/6 inhibitor; Merck Millipore), and 10-μM Y-27632. Approximately 24 h after seeding, equal amounts of Neuronal Differentiation Medium containing 40-ng/mL BDNF (Alomone Labs, Jerusalem, Israel), 40-ng/mL GDNF (Alomone Labs), 20-μM DAPT, 2.0-μM PD0332991, and 10-μM Y-27632 were added to the wells. Half the volume of the medium containing 20-ng/mL BDNF, 20-ng/mL GDNF, 20-μM DAPT, 2.0-μM PD0332991, and 10-μM Y-27632 was changed every other day from day 3 to day 5 of neuronal differentiation. On day 5 of neuronal differentiation, 0.5-mM dibutyryl cAMP was added to the medium. From day 8 to day 15, half the volume of the medium containing 20-ng/mL BDNF, 20-ng/mL GDNF, 20-μM DAPT, 2.0-μM PD0332991, and 10-μM Y-27632 was changed every 2–3 days. After day 15, half the volume of the medium containing 20-ng/mL BDNF and 20-ng/mL GDNF was replaced every 2–3 days until the cells were used for analysis.

### 2.4 Neurosphere formation assay

Expanded NSCs were seeded as a single-cell suspension of 1.0 × 10^4^ cells/well into non-cell-adhesive round-bottomed 96-well PrimeSurface96U plates (Sumitomo Bakelite, Tokyo, Japan) with 200 μL of Neural Expansion Medium containing Y-27632 before being cultured for 5 days. For routine maintenance, half the volume of medium without Y-27632 was changed on even-numbered days. Phase-contrast images of cultured cells were acquired using an IX71 microscope with CellSens software (OLYMPUS, Tokyo, Japan).

### 2.5 WST-8-based cell proliferation assay

Cells were seeded onto a 96-well plate at a density of 1.0 × 10^3^ cells/cm^2^ (undifferentiated human iPSCs) or 3.2 × 10^4^ cells/cm^2^ (expanded NSCs). Cell proliferation was evaluated every 24 h using Cell Count Reagent SF (Nacalai Tesque, Kyoto, Japan) according to the manufacturer's instructions. Briefly, Cell Count Reagent SF was added to each well and cells were incubated for 2 h at 37 °C. Subsequently, viable cells were assessed by measuring the optical density at 450 nm.

### 2.6 Genomic DNA sequencing

Genomic DNA was extracted from iPSCs using a DNeasy Blood and Tissue Kit (QIAGEN, Hilden, Germany). Extracted genomic DNA was amplified by PCR using Platinum Taq DNA Polymerase High Fidelity (Thermo Fisher Scientific); the resultant PCR products were purified using the Wizard SV Gel and PCR Clean-Up System (Promega, WI, USA). Sequencing of the purified PCR-amplified products was outsourced to Eurofins Genomics Inc. (Tokyo, Japan). The primer sets used for this assay are listed in Table S1.

### 2.7 RNA isolation, RT-PCR, and quantitative PCR

Total RNA was extracted from cells using a NucleoSpin RNA Plus XS Kit (Macherey-Nagel GmbH & Co., KG Düren, Germany). From this total RNA, 500 ng was used for reverse transcription, which was achieved using ReverTra Ace qPCR RT Master Mix with gDNA Remover (TOYOBO Co. Ltd., Osaka, Japan). RT-PCR was performed with GoTaq Green Master Mix (Promega), whereas SYBR Green intercalation quantitative PCR was performed with PowerUp SYBR Green Master Mix (Thermo Fisher Scientific). The cDNA synthesized from qPCR Human Reference Total RNA (Takara Bio, Shiga, Japan) was used as a calibrator. The expression of *β-ACTIN* was used as an endogenous control. The primer sets used for this assay are listed in Table S2.

### 2.8 Protein isolation and western blot analysis

Protein was first extracted from cells using M-PER Mammalian Protein Extraction Reagent (Thermo Fisher Scientific) supplemented with cOmplete Mini Protease Inhibitor Cocktail (Merck Miliipore) and PhosSTOP (Merck Millipore) and then incubated on ice for 20 min. Insoluble debris was removed by centrifugation at 13,000*g* and 4 °C for 15 min. The cell lysates were then boiled at 65 °C for 15 min in NuPAGE LDS Sample Buffer (Thermo Fisher Scientific). Protein samples were quantified using a Pierce 660-nm Protein Assay Kit (Thermo Fisher Scientific). An aliquot of the lysates was separated with electrophoresis and transferred to a PVDF membrane using the Trans-Blot Turbo Transfer System (Mini-PROTEAN TGX Precast Gel and Trans-Blot Turbo Mini PVDF Transfer Pack: Bio-Rad, Hercules, CA, USA). After blocking with 5% (*w*/*v*) nonfat dry milk and 0.1% Tween 20 in Tris-buffered saline (TBS-T), the membranes were incubated at 4 °C overnight with primary antibodies against Anti-Niemann−Pick C1 Antibody (Abcam, Cambridge, UK), LC3A/B (D3U4C) XP Rabbit mAb [Cell Signaling Technologies (CST), Danvers, USA], Anti-p62/SQSTM1 (Human) mAb (MBL, Nagoya, Japan), NRF2 (D1Z9C) XP Rabbit mAb (CST), KEAP1 (D6B12) Rabbit mAb (CST), and β-Actin Antibody (CST) diluted in 5% (*w*/*v*) BSA/TBS-T or in 5% (w/v) nonfat dry milk/TBS-T. These membranes were further incubated with “Anti-rabbit IgG, HRP-linked secondary antibody” (CST) or “Anti-mouse IgG, HRP-linked secondary antibody” (CST) diluted in 5% (w/v) nonfat dry milk/TBS-T. To detect specific signals, the membranes were then incubated in ECL Select Western Blotting Detection Reagent (Cytiva: GE Healthcare, Amersham, UK) and bands were visualized using an ImageQuant LAS 500 system (Cytiva). Quantitative analysis was performed using ImageQuant TL software (Cytiva).

### 2.9 Immunofluorescence staining

Cells were fixed in 4% paraformaldehyde (PFA) for 15 min at room temperature (RT), rinsed with DPBS, and then permeabilized with 0.5% Triton X-100 for 15 min at RT. These samples were subsequently incubated with Image-iT FX signal enhancer (Thermo Fisher Scientific) for 30 min at RT. To prevent nonspecific binding of primary antibodies, samples were also incubated with 5% Normal Goat Serum (Vector Laboratories, Burlingame, USA) in DPBS containing 0.3% Triton X-100 for 1 h at RT. After washing with DPBS, samples were incubated at 4 °C overnight with primary antibodies against the following: Anti-Human OCT4 (StemCell Technologies, Vancouver, Canada); Nanog (D73G4) XP Rabbit mAb (CST); LAMP1 (D2D11) XP Rabbit mAb (CST); Anti-NESTIN Antibody, Human-specific (Merck Millipore); Anti-Musashi-1 Antibody (Merck Millipore); Anti-Tubulin Antibody, beta III isoform (Tuj1: Merck Millipore); and MAP2 Polyclonal Antibody (Thermo Fisher Scientific) [all diluted in DPBS containing 0.3% Triton X-100 and 1% (*w*/*v*) BSA]. The following day, samples were incubated at RT for 2 h with Alexta Fluor 488, 555, and 633-conjugated secondary antibodies. After washing with DPBS, samples were counterstained and mounted using ProLong Diamond Antifade Mountant with DAPI (Thermo Fisher Scientific). For immunostaining of cell membranes, DyLight 550-conjugated SSEA-4 Antibody (Stemgent, Cambridge, USA) or a Neurite Outgrowth Staining Kit (Thermo Fisher Scientific) were used with Hoechst 33342 (Thermo Fisher Scientific) diluted in DPBS containing 2% (*w*/*v*) BSA. After again washing with DPBS, samples were mounted using ProLong Diamond Antifade Mountant (Thermo Fisher Scientific). The preparations were then imaged using a Zeiss LSM-880 confocal laser-scanning microscope (Carl Zeiss AG, Oberkochen, Germany) with 10×, 20×, 40×, and 63× objective lenses.

### 2.10 Lipid-droplet analysis

Cells were fixed in 4% PFA for 15 min at RT and then rinsed with DPBS. These samples were subsequently incubated with Lipi-Green (Dojindo, Kumamoto, Japan) and Hoechst 33342, diluted in DPBS containing 1× DPBS (+) Preparation Reagent (Ca/Mg Solution; Nacalai Tesque) and 2% (w/v) BSA, for 2 h at RT. After washing with DPBS, the samples were mounted using ProLong Diamond Antifade Mountant. The preparations were then imaged using a Zeiss LSM-880 confocal laser-scanning microscope with 40× or 63× objective lenses.

### 2.11 Electron microscopy

Cells were fixed in 2% glutaraldehyde and 0.1-M phosphate buffer (pH 7.4), postfixed in 1% osmium tetroxide, and then dehydrated with an ascending ethanol series. The cells were subsequently embedded in epoxy resin before ultrathin sections were produced and double-electronically stained with uranyl acetate and lead citrate. Morphological changes in the organelles of these samples were observed using a Hitachi H-7500 transmission electron microscope (Hitachi, Tokyo, Japan).

### 2.12 Basic image analysis

Quantitative analysis of microscopic image data, such as the size of neurospheres, numbers of cells, and neurite network density, was performed using the open-source image-processing software FIJI (Image*J* ver. 1.53c; National Institutes of Health, MD, USA). To analyze the size of neurospheres, phase-contrast images were acquired with a 4× objective using an IX71 microscope and CellSens software. Brightness, contrast, and threshold were adjusted to enhance sphere outlines, and then the area, circularity, roundness, and Feret's diameter of spheres were measured using the Image*J*. To analyze the number of cells and neurite network density, fluorescent staining images were acquired with a 10× objective using a Zeiss LSM-880 confocal laser-scanning microscope. The original images were converted into a 16-bit images before brightness, contrast, and threshold were adjusted to ensure all cell nuclei and/or neurites could be recognized. Subsequently, the DAPI-stained images were further converted into binary images for cytometric analysis; the number of cell nuclei was counted using the “Analyze Particles” function in Image*J*. Using Tuj1-stained images, the area fraction (%area) occupied by neurites was also measured using Image*J*. Neuronal network density was determined as the TUJ1-positive %area divided by the number of DAPI-positive cell nuclei.

### 2.13 Statistical analysis

Results were compared using two-tailed multiple *t*-tests with ANOVA followed by Tukey's multiple comparisons test. All data are expressed as means ± the standard errors of means (SEMs). *P* values <0.05 were considered statistically significant.

## 3 Results

### 3.1 Characterization of human iPSCs

**Fig. 1:**
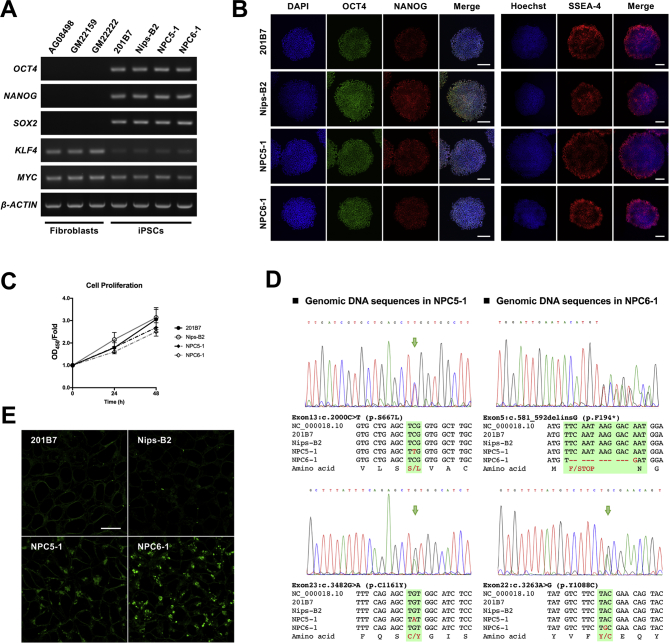
Human iPSCs derived from patients with NPC and *NPC1* mutations show lipid accumulation but do not affect pluripotency and cell proliferation potential. (A) RT-PCR analysis of *OCT4*, *NANOG*, *SOX2*, *KLF4*, and *MYC* gene expression in representative samples of iPSCs (201B7 and Nips-B2 were derived from healthy subjects; NPC5–1 and NPC6–1 were derived from patients with NPC). Fibroblasts from healthy subjects (AG08498, GM22159, and GM22222) were used as negative controls for pluripotency markers and *β-ACTIN* was used as loading control. (B) Representative images of immunostaining for pluripotency markers. Membrane-permeabilized iPSCs were stained with anti-OCT4 antibody (green), anti-NANOG antibody (red), and DAPI (blue), whereas iPSCs without permeabilization were stained with anti-SSEA-4 antibody (red) and Hoechst 33342 (blue). Scale bar = 200 μm. (C) The proliferative capacity of iPSCs was assessed in the logarithmic growth phase by a WST-8-based cell proliferation assay. Data are for fold increases relative to levels at the beginning of the assessment (*N* = 4, mean ± SEM). (D) Mutation analysis of the *NPC1* gene in NPC patient-derived iPSCs. Genomic DNA was extracted from iPSCs, amplified by PCR, subjected to sequencing, and sequences were aligned using ClustalW. (E) Representative images of staining for lipid droplets; iPSCs without permeabilization were stained with Lipi-Green (green). Scale bar = 20 μm.

To verify the pluripotency of healthy control iPSCs (201B7 and Nips-B2) and NPC patient-derived iPSCs (NPC5–1 and NPC6–1), we evaluated the expression levels of *OCT4*, *NANOG*, *SOX2*, *KLF4*, and *MYC* genes, i.e., a set of pluripotent markers, via RT-PCR. Similar to the on-feeder culture conditions, the expression levels of these pluripotent markers was comparable among all iPSCs (Fig. 1A). Furthermore, according to immunofluorescence (IF) staining against OCT4, NANOG, and SSEA-4 and consistent with the gene expression results, the pluripotent markers were uniformly expressed in all iPSCs (Fig. 1B). Additionally, a WST-8 assay showed that no significant difference existed in the cell proliferation ratio among these iPSCs (Fig. 1C). In analysis of the *NPC1* gene mutations in each iPSC type, we found that NPC patient-derived iPSCs had heterozygous mutations consistent with those reported by Soga et al. [30], i.e., c.2000C > T (p.S667L) and c.3482G > A (p.C1161Y) mutations were observed in NPC5–1, whereas c.581_592delinsG (p.F194*) and c.3263A > G (p.Y1088C) mutations were observed in NPC6–1 (Fig. 1D). Since NPC1 mutations result in intralysosomal accumulation of unesterified cholesterol, sphingolipids, and glycolipids in cells [[15], [16], [17]], we evaluated lipid accumulation levels using Lipi-Green (which detects intracellular lipid droplets) and characterized iPSCs derived from patients with NPC. While 201B7 and Nips-B2 showed only a small number of Lipi-Green-positive cells, both NPC5–1 and NPC6–1 showed high levels of lipid accumulation, suggesting that the NPC patient-derived iPSCs reflected the cellular phenotype of NPC (Fig. 1E).

### 3.2 Morphological and biochemical analyses of NSCs derived from iPSCs

**Fig. 2:**
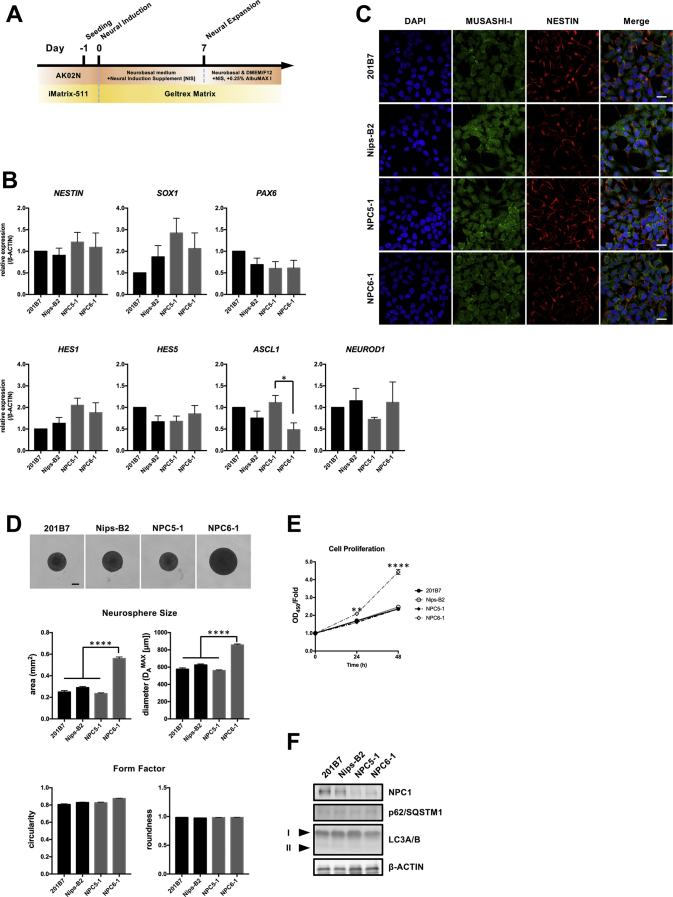
Accelerated cell proliferation shown by NSCs differentiated from NPC patient-derived iPSCs is not common in NPC disease. (A) Protocol for iPSC differentiation into NSCs. (B) qPCR analysis of neural stem/progenitor, radial glia, and immature neuron markers in NSCs. Run data were analyzed using the ΔΔCt method with *β-ACTIN* as the normalizing gene. Data are expressed as fold increases relative to levels in 201B7. *P < 0.05 (ANOVA followed by Tukey's multiple comparisons test; *N* = 4, mean ± SEM). (C) Representative images of immunostaining for neural stem/progenitor markers. Membrane-permeabilized NSCs were stained with anti-MUSASHI-I antibody (green), anti-NESTIN antibody (red), and DAPI (blue). Scale bar = 20 μm. (D) Representative phase-contrast images of healthy control and NPC patient neurospheres (scale bar = 200 μm). Self-renewal ability is characterized by the area (mm^2^), diameter (D_A_^MAX^: μm), circularity, and roundness of each neurospheres. ****P < 0.0001 (ANOVA followed by Tukey's multiple comparisons test; *N* = 4, mean ± SEM). (D) The proliferative capacity of NSCs were assessed in the logarithmic growth phase using a WST-8-based cell proliferation assay. Data represent fold increases relative to levels at the beginning of the assessment. **P < 0.01 and ****P < 0.0001 at 24 h and 48 h, respectively (ANOVA followed by Tukey's multiple comparisons test; *N* = 3, mean ± SEM). (E) Western blot analysis of NPC1, p62/SQSTM1, and LC3A/B protein expression in representative NSCs. The expression level of β-ACTIN was used as a loading control. Arrowheads indicate the LC3-I and -II forms of LC3A/B, respectively. Similar results were obtained in four independent samples.

**Fig. 3:**
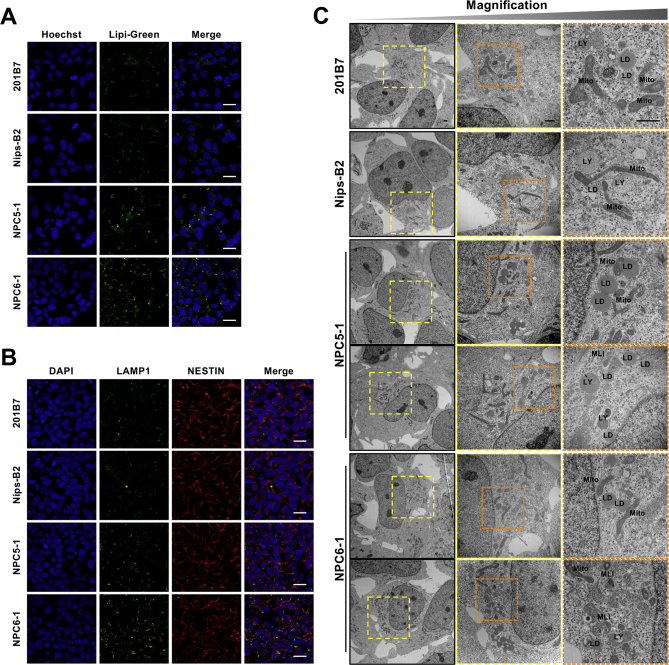
NPC patient-derived NSCs show accumulation of lipid droplets and lysosomes as well as increased lipid droplets–mitochondria contact. (A) Representative images of staining for lipid droplets. NSCs cultured in 0.25% AlbuMAX I-containing medium were stained with Lipi-Green (green) and Hoechst 33342 (blue). Scale bar = 20 μm. (B) Representative images of immunostaining for lysosomes. NSCs cultured in 0.25% AlbuMAX I-containing medium were stained with anti-LAMP1 antibody (green), anti-NESTIN antibody (red), and DAPI (blue). Scale bar = 20 μm. (C) Ultrastructural pathology in NPC patient-derived NSCs. Magnified images of the organelles are shown in the middle and right panels (colored boxes with dashed lines indicate the magnifications). Abbreviations: LY, lysosome; Mito, mitochondria; MLI, multilamellar inclusion; LD, lipid droplets. Scale bar = 1 μm.

To investigate the neuronal phenotype and pathological mechanism of NPC in vitro in a cell-based model, iPSCs from healthy controls and patients with NPC were differentiated into the neural lineage (Fig. 2A). Gene expressions of major neural stem/progenitor markers (*NESTIN*, *SOX1*, and *PAX6*), radial glia markers (*HES1* and *HES5*), and immature neuron markers (*ASCL1* and *NEUROD1*) were then evaluated by qPCR. Although we slightly modified the medium composition used for the maintenance of NSCs as reported by Yu et al. and Soga et al. [28,30], we consistently found no significant difference between the healthy and diseased cell lines (Fig. 2B). Consistent with gene expression results, IF staining revealed that neural stem/progenitor markers (NESTIN and MUSASHI-I) were uniformly expressed in all NSCs (Fig. 2C), suggesting that NPC patient-derived iPSCs had levels of neural differentiation potential that were almost equal to those of healthy control cells. Furthermore, these NSCs were equally capable of forming neurospheres and only NPC6–1-derived NSCs showed a significant increase in sphere size with increasing cell proliferation capacity (Fig. 2D, E). On the other hand, results from our western blot analysis differed from the results of previous studies [30], which have shown that autophagy abnormalities in many cells and tissues (including fibroblasts and NSCs) exist in patients with NPC. Indeed, accumulation of p62/SQSTM1 and LC3-II, an indicator of autophagy induction, was not observed under the present experimental conditions. However, we did detect a significant decrease in NPC1 protein in response to the genetic mutations (Fig. 2F). To determine whether these NSCs reflected NPC pathology, we evaluated the accumulation of lipid droplets and LAMP1-positive lysosomes; NPC patient-derived NSCs showed intracellular accumulation of lipid droplets and LAMP1-positive lysosomes, albeit in smaller amounts than those previously reported (Fig. 3A, B) [28,30]. Furthermore, thin-sectioning electron microscopy (TEM) analysis showed some hallmarks of NPC. Similar to the findings of Yu et al. [28], the enlarged lysosomes containing lamellar structures were frequently observed in NPC patient-derived NSCs (Fig. 3C). In addition, however, we found that some lipid droplets and mitochondria made contact in NPC patient-derived NSCs but did not do so in healthy control cells (Fig. 3C). Consistent with IF staining, lipid droplets were also observed in healthy control-derived NSCs, but they were detected less frequently than in NPC patient-derived NSCs. These results indicate that NPC patient-derived NSCs reflect a few pathologies, such as accumulation of lipids and lysosomes. In addition, NSCs in immature stage may not accurately reflect the neuropathology. Therefore, we chose to use the neurons differentiated from these NSCs to analyze neuropathology in detail.

### 3.3 Neuropathological analysis of neurons differentiated from iPSCs-derived NSCs

**Fig. 4:**
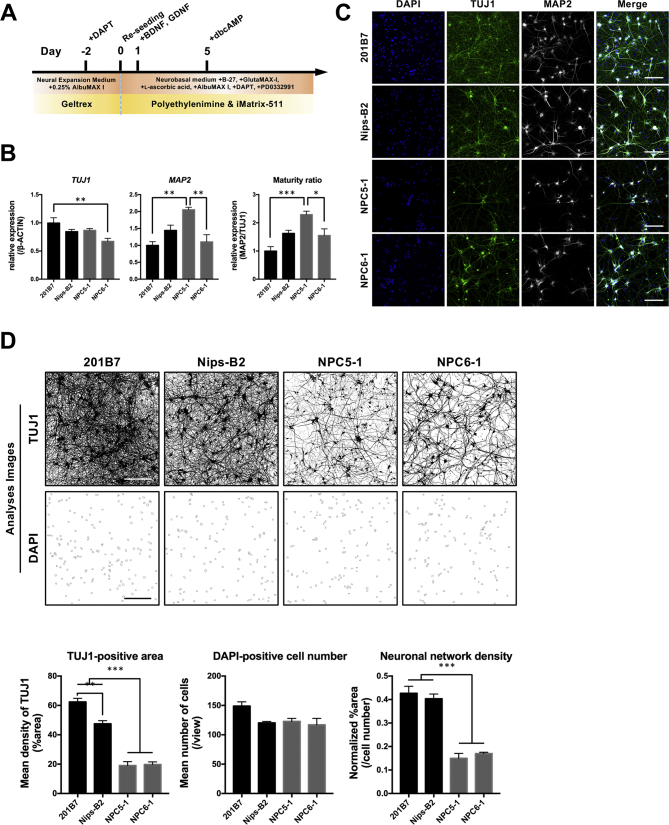
NPC patient-derived neurons show reduced neuronal network density. (A) Protocol for differentiation of iPSCs-derived NSCs into neurons. (B) qPCR analysis of neuronal markers in neurons. Run data were analyzed by the ΔΔCt method; cDNA from qPCR Human Reference Total RNA was used as a calibrator and *β-ACTIN* expression was used for normalization. Data are expressed as fold increases compared with levels in 201B7. *P < 0.05, **P < 0.01, and ***P < 0.001 (ANOVA followed by Tukey's multiple comparisons test; N = 4, mean ± SEM). (C) Representative images of immunostaining for neuronal markers. Membrane-permeabilized neurons were stained with anti-TUJ1 antibody (green), anti-MAP2 antibody (white), and DAPI (blue). Scale bar = 100 μm. (D) Representative analysis images of TUJ1-positive neurites and DAPI-positive nuclei (scale bar = 200 μm). Neuronal network density was determined as TUJ1-positive %area divided by the number of DAPI-positive cell nuclei (the two latter variables were quantified using FIJI). **P < 0.01, ***P < 0.001 (ANOVA followed by Tukey's multiple comparisons test; N = 4, mean ± SEM).

**Fig. 5:**
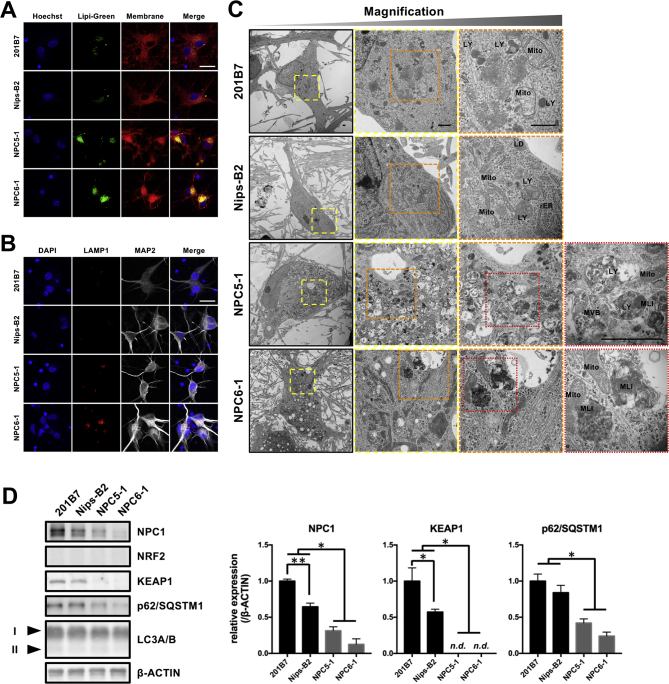
NPC patient-derived neurons show definite pathological features represented by accumulations of lipid droplets, lysosomes, lamellar inclusions and disruption of the p62/SQSTM1−KEAP1−NRF2 axis. (A) Representative images of staining for lipid droplets and cell membranes. Neurons cultured in 0.25% AlbuMAX I-containing medium were stained with Lipi-Green (green), Cell Membrane Stain (red), and Hoechst 33342 (blue). Scale bar = 20 μm. (B) Representative images of immunostaining for lysosomes and MAP2. Neurons cultured in 0.25% AlbuMAX I-containing medium were stained with anti-LAMP1 antibody (red), anti-MAP2 antibody (white), and DAPI (blue). Scale bar = 20 μm. (C) Ultrastructural pathology in NPC patient-derived neurons. Magnified views of the organelles are shown in the second to fourth right panels (colored boxes with dashed lines indicate the magnifications). Abbreviations: LY, lysosome; Mito, mitochondria; MLI, multilamellar inclusion; MVB, multivesicular body; LD, lipid droplets. Scale bar = 1 μm. (D) Western blot analysis of NPC1, NRF2, KEAP1, p62/SQSTM1, and LC3A/B protein expression in representative neuron samples. The expression level of β-ACTIN was used as a loading control. Arrowheads indicate the LC3-I and -II forms of LC3A/B, respectively. Quantitative data are normalized against β-ACTIN and expressed as fold increases relative to levels in 201B7. *P < 0.05 and **P < 0.01 (ANOVA followed by Tukey's multiple comparisons test; *N* = 3, mean ± SEM).

For detailed analysis of the pathological changes in the neurons of patients with NPC, NSCs derived from iPSCs were terminally differentiated into mature neurons and their characteristics were analyzed after 4 weeks of culture (Fig. 4A). Terminal neurodifferentiation resulted in morphological changes, such as the projection of neurites and formation of neuronal networks, as well as the expression of major neuronal marker genes (*TUJ1* and *MAP2*). Although each cell line showed different levels of gene expression and cell maturation, there was no consistent difference between the healthy control groups and patient groups (Fig. 4B). IF staining also demonstrated that all differentiated cells showed a neuron-like morphology with expression of TUJ1, whereas other proliferating cells were not observed in this culture (Fig. 4C). Therefore, the cell population after terminal neurodifferentiation was considered not to have contained any residual NSCs, differentiated astrocytes, and/or oligodendrocytes, which have proliferative properties unlike neurons. Although the NPC patient-derived neurons and healthy control equivalents had almost equal levels of neurodifferentiation potentially, the ability of the NPC patient-derived neurons to form complex neuronal networks appeared to have been diminished. To assess the complexity of these neuronal networks, the ratio of TUJ1-positive areas to the number of cells was calculated in given images; the formation of neuronal networks was found to be significantly reduced in the NPC patient group (Fig. 4D). This reduced ability to form neuronal networks may therefore be a commonly detected NPC pathology that is independent of cell line. Furthermore, IF staining and TEM analysis showed accumulations of cholesterol and glycolipids shown as lipid droplets and increased numbers of lysosomes with massive inclusion bodies in neurons differentiated from NPC patient-derived iPSCs, which reflects the typical NPC pathology (Fig. 5A−C). Specifically, IF analysis revealed that greater accumulations of lipid droplets and LAMP1-positive lysosomes existed in MAP2-positive mature neurons differentiated from NPC patient-derived iPSCs relative to less accumulations in healthy controls (Fig. 5A, B). Additionally, TEM analysis revealed that NPC patient-derived neurons had numerous vacuoles and enlarged lysosomes containing lamellar structures and dense bodies, indicating generalized endocytic dysfunction and storage of multiple lipid species (Fig. 5C). Therefore, these findings suggest that reduced neuronal network formation in NPC patient-derived neurons may be based on the pathology of NPC disease.

Since our neuropathological model of NPC disease had showed a significant decrease in neuronal network formation and excessive accumulations of lysosomes, we analyzed the p62/SQSTM1−KEAP1−NRF2 axis, as a representative of the autophagic pathway and oxidative stress response involved in neurodegeneration [33]. Under nonoxidative stress conditions, NRF2 is homeostatically ubiquitinated by the KEAP1/CUL3/RBX1 E3 ubiquitin ligase complex and degraded by the 26S proteasome [34]. When KEAP1 senses electrophilic or oxidative stress, it stops the ubiquitination of NRF2, which leads to the activation of NRF2. On the other hand, p62/SQSTM1, which is involved in the induction of selective autophagy, competes with the binding of NRF2 to KEAP1 [35]. Therefore, abnormalities in autophagy accompanied by an accumulation of p62/SQSTM1 indirectly activate NRF2. Contrary to our expectations, western blot analysis revealed decreased protein expression of p62/SQSTM1 and KEAP1 in the NPC patient group. Moreover, there was no accumulation of NRF2 or increase in LC3-II in any of the cells (Fig. 5D). These results suggest that the p62/SQSTM1−KEAP1−NRF2 axis is disrupted in mature neuronal cells generated from NPC patient-derived iPSCs independent of the autophagy pathway.

## 4 Discussion

The pathology of NPC disease includes intralysosomal accumulation of unesterified cholesterol, sphingolipids, and glycolipids associated with abnormal intracellular lipid transport. In this study, we newly found that there was no definite intracellular lipid accumulation in NPC patient-derived iPSCs during immature cell proliferation stage. In other words, these iPSCs show stable cell proliferative capacity while maintaining an undifferentiated state similar to that of healthy control-derived iPSCs. In addition, NPC patient-derived iPSCs did not show any characteristic lysosomal accumulation (data not shown), nor any significant differences between these iPSCs and healthy control-derived iPSCs, which may have been due to the immaturity of the intracellular organelles. Therefore, we surmised that a clearer picture of the pathological features in NPC disease would be determined by analyzing cells after differentiation.

Cells obtained by induction of neural differentiation showed gene and protein expression of markers that indicated they were NSCs. Although Sung et al. [32] reported impairment of self-renewal and neuronal differentiation in NPC patient-derived NSCs, we found no such as defects in the present study. We speculate that this difference may be due to the method of differentiation into NSCs. In the work of Sung et al., NSCs were directly induced from NPC patient-derived fibroblasts carrying NPC1^P237S/I1061T^ or NPC1^I1061T/I1061T^ mutations by expression of *SOX2* and *HMGA2* using retroviral vectors. In contract, we induced NSCs from iPSCs carrying NPC1^S667L/C1161Y^ or NPC1^F194⁎/Y1088C^ using small-molecule compounds. The direct induction of NSCs from fibroblasts without establishment of iPSCs can reproduce the patient's pathology at the time of biopsy. Since iPSCs undergo reprogramming with elimination and reconfiguration of epigenetic marks, including DNA methylation, the patient's pathology is also expected to be initialized. Therefore, we hypothesized that NSCs in the early stages of development would not show abnormalities, whereas the NSCs in the adult brain would be impaired, as reported by Sung et al. In support of this hypothesis, Seo et al. [36] reported that NSCs derived from the lateral subventricular zone of NPC model mice showed enhanced c-Jun N-terminal kinase activity and DNA damage, which led to impairment of neurogenesis. Although it is unclear whether similar impairment occurs in actual patients with NPC disease, these findings provide potential insights into the pathogenesis of the disease.

To maintain stemness of NSCs derived from iPSCs, serum-free medium and/or serum substitutes are commonly used to culture NSCs. However, neural stem cell medium containing 10% FBS has frequently been used to demonstrate disease phenotypes in NSCs derived from patients with NPC [28,30]. Since FBS contains hormones and growth factors, this approach introduces concerns about unexpected differentiation and impaired stemness. To address such concerns, we tested a new method by which to evaluate the phenotype of NPC disease in the NSC state: we used Neural Expansion Medium supplemented with 0.25% AlbuMAX I Lipid-Rich BSA as a lipid donor. NSCs maintained in 0.25% AlbuMAX I Lipid-Rich BSA-containing medium stably expressed neural stem/progenitor markers regardless of the number of passages. In addition, we demonstrated that the accumulation of lipid droplets specifically occurred in the NPC patient group. Therefore, we suggest that this culture method represents a model that can reproduce the pathogenesis of NPC disease without concern for the reduction of stemness potentially caused by FBS. FL and electron microscopy revealed characteristic lysosomal accumulation, such as lamellar structures, in NSCs derived from patients with NPC; nevertheless, the frequency at which such accumulation occurred seemed to be lower than that of terminally differentiated cells, such as fibroblasts, macrophages, and hepatocytes [[37], [38], [39]]. A new finding in the current study was that intracellularly accumulated lipid droplets and mitochondria made contact in NPC patient-derived NSCs. Höglinger et al. [40] reported that the interaction between NPC1 and Gramd1b is involved in the tethering late endosomes/lysosomes to the ER to regulate cholesterol egress; this tethering tended to shift from the ER to the mitochondria in NPC1-deficient cells. For the first time, we have shown that lipid droplets may also be tethered to mitochondria in NSCs derived from patients with NPC. Although immunoelectron microscopy analysis will be required to verify the direct binding of lipid droplets to mitochondria, we speculate that this tethering may also be involved in the supply of lipids to mitochondria. Such an excessive supply of lipids to mitochondria would be a likely cause of mitochondrial dysfunction. Indeed, Soga et al. reported decreased ATP production in NSCs derived from patients with NPC, supporting the occurrence of mitochondrial dysfunction in NPC disease [19,30]. Contrary to other reports [30,41], however, we did not obtain observe abnormalities of autophagy such as accumulation of autophagosomes, increased expression of p62/SQSTM1, and conversion of LC3-I to -II in NSCs derived from patients with NPC. In contrast, Sarkar et al. found that stimulation of autophagy by nutrient starvation decreased p62/SQSTM1 and LC3-II/autophagosome levels in NPC patient-derived fibroblasts due to increased autophagic flux; furthermore, the excessive accumulation of p62/SQSTM1 in these cells was more clearly observed under normal culture with 10% FBS [30,41]. Therefore, we postulate that because autophagic flux was increased by sustained nutrient starvation, the accumulation of p62/SQSTM1 and LC3-II/autophagosomes was not observed under our culture conditions. In addition, we would expect that the excessive accumulation of p62/SQSTM1 to have been detected if NPC patient-derived NSCs were maintained in 10% FBS-supplemented medium, as previously reported [30].

In the present study, fluorescent staining of NPC patient-derived neurons showed that accumulation of lipid droplets and lysosomes consistently occurred from the undifferentiated iPSCs state. Moreover, the accumulation of characteristic lysosomes containing lamellar structures and numerous vacuoles were remarkably observed in NPC patient-derived neurons as the maturation of the organelle occurred, as shown in TEM analysis. These results support our suggestion that an accurate disease phenotype can only be reconstructed by differentiating to mature cells. Using this neuropathological model, we discovered that neuronal network formation is significantly decreased in patients with NPC. Given that there was no consistent change in *TUJ1* and *MAP2* gene expression levels between the healthy and NPC patient groups, we surmised that decreased in neuronal network formation was not due to abnormal cell differentiation, maturation, or developmental delay but may instead have been related to neurodegeneration in mature neuronal cells. Furthermore, we reported for the first time that levels of p62/SQSTM1 and KEAP1 proteins were reduced in neurons differentiated from NPC patient-derived iPSCs. However, an accumulation of NRF2 or conversion of LC3-I to -II did not occur in any of the neurons differentiated from iPSCs. Since the expression of several NRF2 response genes, such as glutathione peroxidase 4, Mn-superoxide dismutase, and heme oxygenase 1, was not promoted (data not shown), we conclude that the NRF2 pathway was not activated by the decrease in KEAP1, even in neurons differentiated from NPC patient-derived iPSCs. This raises the possibility that the entire p62/SQSTM1−KEAP1−NRF2 axis was disrupted as opposed to decreases in protein expression limited to p62/SQSTM1 and KEAP1. The loss of p62/SQSTM1 has recently been associated with neurodegeneration and accumulation of hyperphosphorylated tau [42]. Furthermore, the expression levels of p62/SQSTM1 are known to be decreased in several neurodegenerative diseases owing to age-related oxidative damage to DNA [43,44]. In addition, previous transcriptomic and proteomic analyses of neurons differentiated from p62/SQSTM1-deficient cells suggested that p62/SQSTM1 regulates the metabolic shift from aerobic glycolysis to oxidative phosphorylation, which is essential for neuronal differentiation and maturation [45]. In agreement with these collective studies, we found that neuronal network formation was impaired and p62/SQSTM1 protein levels decreased in neurons differentiated from NPC patient-derived iPSCs. Therefore, we conclude that the reduction of p62/SQSTM1 protein in these neurons was one of the causes of impaired neuronal network formation.

## 5 Conclusions

The main findings of this study can be summarized as follows: (1) lipid droplets which are characteristic of NPC disease accumulated in both undifferentiated NPC patient-derived iPSCs and derived NSCs, but other abnormalities in differentiation potential, proliferative capacity, and autophagic activity were not detected; (2) with terminal differentiation into mature neurons, more definite neuropathological features were clearly observed in NPC disease as demonstrated by LAMP1 and lipid droplets staining, and TEM analysis; (3) disruption of the p62/SQSTM1−KEAP1−NRF2 axis occurred in neurons differentiated from NPC patient-derived iPSCs. This phenomenon may decrease to the neuronal network formation. To increase confidence in the observed phenotypes, it would be necessary to correct the NPC1 point mutant by genome editing to create a isogenic controls; by applying the same culture conditions and differentiation protocol to the NPC1 mutant and the paired isogenic controls, we could be more confident that the phenotypic changes are solely attributable to the NPC1 mutation. Nevertheless, we believe that use of our neuropathological model of NPC disease could lead to new insights into disease pathology and the discovery of novel drug targets.

## Funding

This work was supported by Grant-in-Aid for Science Research (KAKENHI) 17K10067 and 19K16382 from the 10.13039/501100001700Ministry of Education, Culture, Sports, Science and Technology of Japan (http://www.jsps.go.jp/english/e-grants/). This work was also supported by 10.13039/100009619AMED under Grant Number JP20ek0109460 and Sanofi Investigator Sponsored Study (No. SGZ-2019-12859). The funding bodies played no role in the study design, data collection and analysis, decision to publish, or preparation of the manuscript.

## Declaration of Competing Interest

Yoshikatsu Eto has received Y. E. Research grants from Sanofi Co. and Actelion Pharmaceuticals. Other authors has no conflict of interest.

## Appendix A Supplementary data

Supplementary material Image 1
