# Supplementary material for: A neuropathological cell model derived from Niemann−Pick disease type C patient-specific iPSCs shows disruption of the p62/SQSTM1−KEAP1−NRF2 Axis and impaired formation of neuronal networks
Source: Mol Genet Metab Rep. 2021 Jul 24;28:100784. doi: 10.1016/j.ymgmr.2021.100784 (PMC8327345; doi:10.1016/j.ymgmr.2021.100784)
Supplement: Supplementary file 1 — Supplementary material [file mmc1.docx]

**Table S1: Primer sequences for genomic sequencing of *NPC1* coding region.**

| Exon No. | Forward Primer Sequence (5’−>3') | Reverse Primer Sequence (5’−>3') |
| --- | --- | --- |
| 1 | ACGGCCAGAAACCGTTG | CTCTCCATCGCCAGACCAACTTC |
| 2 | TGTTAAGTGCCTGTTGCTGT | AGAGTTTGAGAGTCCGGGATAAG |
| 3 | GAGCCACAGTACCTGTCCTCA | GGAAAGCTGAGCATTACCAGTTCA |
| 4 | AATCGTTCTTGCTGGCCCTATT | TCTGTTAGACCTGCTGACTTTCC |
| 5 | GGGAATCTCTGTTCGTGCC | CTGGACCCACGCAATCC |
| 6 | GATTCCTCTATCAGTGACAAACC | GCTCTGACAAATGAAAGCTC |
| 7 | CAGGTTGGTCTCGATCTCT | CCCTGCTATTCCCACTCTT |
| 8 | GGTGGGAATAGCGCTCATTGAGG | TACCATGACATTCAGCCC |
| 9 | CTTTGTCTTCTGACCC | AGGCTGAGGTATGAG |
| 10 | GTGATTCTGAGCTCCCAAAGGTGAG | GGGAAAGGTACTTAGCCTTCGACAG |
| 11 | GCCCAGAGATACAGTCCATAGCTCC | GGAGGGAGAGCTGTTTAAAGCACC |
| 12 | AGAAAACGTGGCCTTTGTATCGTG | TGCCTAGTGAGACCTTCGAATCC |
| 13 | ACGTTTCTGTTGCCTACATC | TAAGATGTCCACAGCAAGTC |
| 14 | CACAAGGCAGCAAGAAATGG | TCCTTCTTTCTCCAGGCTCA |
| 15 | TGGCCTGTATTAGTGGTTCTCA | CTCATCCAGTCCTTTAGCAGAAGT |
| 16 | GTTTCGTGAGTCTCTTGGGGTTAGAC | ATCCTGACTCTGCCACTTACGG |
| 17 | CCAAGTCACTCAGCCCGTAAGTG | CTGCTAGCCCCACAATCAGGAG |
| 18 | CTGGCACCCTCTTATTCTCCGT | CTGTGGCTTCACCCAGTCGAA |
| 19 | GGAGGAAGGGCACGACTACA | GGTATAAACTGAGGCACGATGCAA |
| 20 | TTGCCTCTCAGGATTGGTATCTGG | GAGAAGAGACGTTCCCATGCAAC |
| 21 | GCAAGACCTGGACTCTCTTGACAC | CCAAATAACCAGCAGTTAGGGATTCTGG |
| 22 | GGGATGTTTCCCAAAGGAGTTTGACC | CAATGCTCGCTCCCTCTATGCC |
| 23 | ATGCTTGGCCTCCTCTAGCAC | GCTGCTTTGTAAGTACAGGATCCAGAC |
| 24 | ATCAATTGGGAGACACGATG | TGCCTCAGGATAGAATTCCC |
| 25 | CAGTCCCACCTACTTGTTAGGCTG | TGAGTTCACAGGCGCTACGTTC |

**Table S2: Primer sequences for PCR.**

| Genes | Forward Primer Sequence (5’−>3') | Reverse Primer Sequence (5’−>3') |
| --- | --- | --- |
| OCT4 | TGAGTAGTCCCTTCGCAAGC | TAGCCAGGTCCGAGGATCAA |
| NANOG | GTATTGTTTGGGATTGGGAGGC | TACGATGCAGCAAATACGAGAC |
| SOX2 | AGCTACAGCATGATGCAGGA | GGTCATGGAGTTGTACTGCA |
| KLF4 | TCTCAAGGCACACCTGCGAA | TAGTGCCTGGTCAGTTCATC |
| MYC | ACTCTGAGGAGGAACAAGAA | TGGAGACGTGGCACCTCTT |
| NESTIN | GGCGCACCTCAAGATGTCC | CTTGGGGTCCTGAAAGCTG |
| SOX1 | GGCCGAGTGGAAGGTCATGT | GCCGGTACTTGTAATCCGGG |
| PAX6 | ACCACACCGGTTTCCTCCTTCACA | TTGCCATGGTGAAGCTGGGCAT |
| HES1 | CCAAAGACAGCATCTGAGCA | CATTGATCTGGGTCATGCAG |
| HES5 | CTCAGCCCCAAAGAGAAAAA | TAGTCCTGGTGCAGGCTCTT |
| ASCL1 | GATGAGTAAGGTGGAGACACTGCG | CCGACGAGTAGGATGAGACCG |
| NEUROD1 | TCCGGAGGCCCCAGG | CGCCCATCAGCCCACTC |
| TUJ1 | CATGGACGAGATGGAGTTCA | TTCGTACATCTCGCCCTCTT |
| MAP2 | CAGAAGTTCAGGCCCACTCT | GGTTTTCCGCTTAACACAGG |
| β-actin | TCCTCCCTGGAGAAGAGCTAC | TCCTGCTTGCTGATCCACAT |
